# A mortality review of tuberculosis and HIV co-infected patients in Mahalapye, Botswana: Does cotrimoxazole preventive therapy and/or antiretroviral therapy protect against death?

**DOI:** 10.4102/phcfm.v10i1.1765

**Published:** 2018-11-15

**Authors:** Stephane Tshitenge, Gboyega A. Ogunbanjo, Andre Citeya

**Affiliations:** 1 Department of Family Medicine and Public Health, University of Botswana, Botswana; 2 Department of Family Medicine and Primary Health Care, Sefako Makgatho Health Sciences University, South Africa; 3 Mahalapye District Health Team, Mahalapye, Botswana

## Abstract

**Background:**

The World Health Organization aims to reduce tuberculosis (TB) mortality rate from 15% in 2015 to 6.5% by 2025.

**Aim:**

This study determined the profile of TB and human immunodeficiency virus (HIV) co-infected patients who died in Mahalapye District, Botswana, while on anti-TB medication and the factors that contributed to such outcome.

**Setting:**

The study was conducted in the Mahalapye Health District in Botswana.

**Methods:**

This was a cross-sectional study that reviewed patient records from the Mahalapye District Health Management Team Electronic Tuberculosis Register from January 2013 to December 2015.

**Results:**

The majority of the TB and HIV co-infected patients were on antiretroviral therapy (ART) (486 [81.63%]) or were initiated cotrimoxazole preventive therapy (CPT) (518 [87.2%]) while taking anti-TB treatment. Seventy-three (13.6%) TB and HIV co-infected patients died before completing anti-TB treatment. Three-quarters (54 [74.4%]) of patients who died before completing anti-TB treatment were on ART, among them two patients who were on ART at least 3 months prior to commencing anti-TB. Also, the majority (64 [87.7%]) of TB and HIV co-infected patients were commenced on CPT prior to death. There was a bimodal density curve of death occurrence in those who did not commence ART and in those who did not commence CPT.

**Conclusion:**

This study established that TB and HIV co-infected patients had a TB mortality of 13.6%. A high mortality rate was observed during the first 3 months in those who did not take ART and during the second and the fifth month in those who did not commence CPT. Further study is needed to clarify this matter.

## Introduction

In 1993, the World Health Organization (WHO) acknowledged that tuberculosis (TB) is a worldwide public health emergency in view to end a long period of neglecting the disease.^1^ TB and human immunodeficiency virus (HIV) co-infection has high prevalence and mortality. WHO policies on collaborative TB and/or HIV activities of 2004 and 2012 had since recommended delivery of TB and HIV services concomitantly for prevention, diagnosis, treatment and care of patients with TB and HIV co-infection.^2,3^ Although Africa counts for only 1 in 10 persons of the world’s population, it carries about 3 in 10 cases of the global burden of TB cases and about 4 in10 cases of TB global mortality.^4^ Botswana has one of the highest incidences of TB in Africa, and TB is probably the most common cause of death in the country.^5^ With a rate of about 6 in 10 patients with active TB being co-infected with HIV, TB also is a leading cause of morbidity and mortality in people living with HIV in Botswana.^6^

The WHO’s target is to reduce TB mortality rates from 15% in 2015 to 6.5% by 2025.^1^ A high proportion of TB and HIV co-infected patients tend to die within the first 3 months after they commenced anti-TB therapy. Later presentation to a health facility, diagnosis processes, opportunistic infections or immune reconstitution syndrome may contribute to such high proportion of TB and/or HIV mortality.^7,8^ Commencing antiretroviral therapy (ART) and cotrimoxazole preventive therapy (CPT) in all HIV-positive TB patients regardless of WHO clinical stage or CD4 cell count are some strategies to reduce TB mortality. In the 2016 updated guidelines, the WHO recommended to start ART no later than eight weeks after starting anti-TB treatment.^4,9^

Studies focusing on TB mortality within the first 3 months are scarce; in the majority of these published studies, the population studied were hospitalised patients.^10^ In 2008, Botswana introduced guidelines for CPT and ART for TB and HIV co-infected patients regardless of their CD4 levels^11^; this was even before the global 2010 recommendation.^9,10^ After implementing such measures, it is crucial to evaluate how Mahalapye Health District (MHD) performed and how far the district is in attaining the 2025 WHO TB mortality target. Also, factors that contribute to TB mortality may enable the health team to make a plan to prevent potential TB deaths. This study determined the profile of TB and HIV co-infected patients who died in MHD, Botswana, while on anti-TB treatment.

## Methodology

### Study design and period

This was a cross-sectional study that reviewed patient data over a period of 3 years from 01 January 2013 to 31 December 2015.

### Study setting

The MHD is located in Mahalapye sub-districts (Central District, Botswana), with an estimated population of 118 875.^12^ It has 46 health facilities (1 district hospital, 1 primary hospital, 15 clinics and 29 health posts) that offer a comprehensive TB service comprising diagnosis, contact tracing, drug supply and direct observation of anti-TB treatment. Botswana has a TB prevalence of 470 per 100 000 people and an HIV prevalence of 18.5%.^13,14^ Both direct observation of therapy and community TB care are provided in the health district. Once TB is diagnosed, the patient is tested for HIV, if HIV status is unknown. For patients with TB and HIV co-infection, CPT is initiated together with anti-TB treatment. For those not yet on ART, it is introduced within the first eight weeks of anti-TB treatment if the CD4 is above 50 cells/mm^3^; for those with CD4 below 50 cells/mm^3^, ART is introduced as soon as the patient is stable.

In 2014, Mahalapye District Hospital established a multidrug resistant tuberculosis (MDR-TB) management site, where patients with MDR-TB are managed. Hospitals have laboratories that perform either microscopy or GeneXpert testing for diagnosis. Data are captured locally (at the health facility) on paper registers and information is sent to the Mahalapye District Health Management Team (M-DHMT) where it is entered into the Electronic Tuberculosis Register (ETR). The MHD is assisted technically in the TB programme by a Botswana–University of Pennsylvania partnership through training and mentoring.

### Study population, sample size and selection

We reviewed all ETR records for patients diagnosed with TB in M-DHMT during the study period. The M-DHMT–ETR recorded a total of 1086 patients who were initiated on -anti-TB therapy from 01 January 2013 to 31 December 2015.

### Data collection, data analysis and procedure

We extracted data from the M-DHMT–ETR that included independent variables such as: health facility, sex, age, type of TB (pulmonary or extra-pulmonary), TB categories (new, retreatment), initial sputum results, duration of anti-TB treatment, HIV status, taking ARV or taking CPT. The dependent variable was ‘died during anti-TB treatment or not’. We defined TB mortality as all-cause mortality before completing anti-TB treatment.^15^

Data were captured using a spreadsheet and summarised as mean ± standard deviation (s.d.) for normally distributed variables and frequency in percentages for binomial and median ± interquartile range if skewed. We used the chi-square test to determine whether there was an association between each independent variable and TB mortality. All statistical analysis was performed using R software, version 3.3.1. The level of significance was set at *p* < 0.05.

### Ethical consideration

We used only records in the ETR and TB facility registers to obtain the data. We obtained ethical approval from the Health Research Unit of the Ministry of Health (HPME 13/18/1 X1 [76]) and the M-DHMT Ethics Committee (MH/DHMT/1/7/7 [25]). We also obtained a waiver of consent from the M-DHMT Ethics Committee, as the study dealt with patient records only. To ensure confidentiality, patient identifiers, such as names and identity numbers, were excluded in the data collection sheet.

## Results

The M-DHMT–ETR had a record of 420 (38.7%) patients in 2013, 370 (34.1%) in 2014 and 296 (27.2%) in 2015. Major health facilities, such as Airstrip Clinic and Xosa Clinic, had more cases during anti-TB treatment (98 and 60, respectively), while the Mahalapye District Hospital recorded 41 patients taking anti-TB treatment at their site. Six hundred and eight (56.0%) were male patients and 478 (44.0%) were female patients, and their median age was 37 (s.d. ±19) years. A total of 124 (11.4%) patients died before completing anti-TB treatment. Four cases were classified as MDR-TB bacteria, of which two were HIV co-infected.

Of all the cases, 594 (54.7%) were HIV-positive and 392 (36.1%) were HIV-negative. The HIV status was not recorded in 100 (9.2%) cases in the M-DHMT–ETR.

**FIGURE 1:**
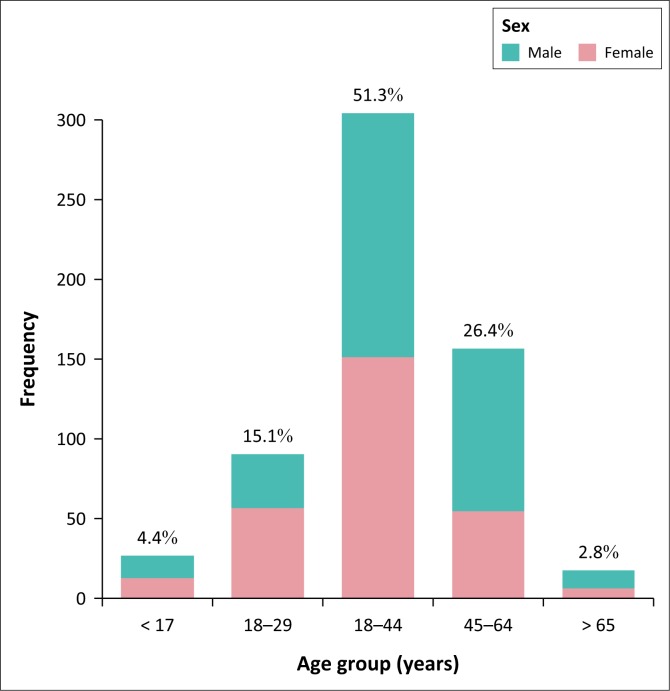
Age group and sex distribution in patients with tuberculosis and HIV co-infection cases, Mahalapye Health District, January 2013 to December 2015.

In all, 475 (80.0%) TB and HIV co-infected patients were in the TB category of new cases. As illustrated in Figure 1, the majority of TB and HIV co-infected patients were in the age group 30–40 years (305 [51.3%]) or in the age group 45-64 years (127 [26.4%]). Pulmonary TB was diagnosed in 463 (77.9%) cases, while 108 (18.2%) cases were in the extra-pulmonary category in TB and HIV co-infection. Of the pulmonary TB cases, 325 (70.2%) were bacteriologically confirmed by microbiology or GeneXpert, and in 138 (29.8%) cases the diagnosis was made clinically (Table 1). The majority of the TB and HIV co-infected patients were on ART (486 [81.63%]) or were initiated on CPT (518 [87.2%]) while taking anti-TB treatment.

**TABLE 1:** Tuberculosis and HIV co-infection cases’ categories in Mahalapye Health District, January 2013 to December 2015.

Category	*n*	%		Pulmonary TB (*N* = 463)			Extra-pulmonary TB	On ART while on anti-TB‡	On CPT while on anti-TB§	Death while on anti-TB
				Bact. confirmed†	Clinically diagnosed					
New	475	80.0		253	110		95	377	411	58
Relapse	109	18.4		64	26		13	96	97	13
Default	6	0.10		4	2		-	5	6	2
Failure	4	0.06		4	-		-	4	4	0
**Total, *n***	**594**	-		**325**	**138**		**108**	**486**	**518**	**73**
**%**	-	-		**70.2**	**29.8**		**18.2**	**81.8**	**87.2**	**12.3**

TB, tuberculosis; Bact., bacteriologically; N, number; ART, antiretroviral therapy; CPT, cotrimoxazole preventive therapy.

† , Bacteriologically confirmed by microscopy or GeneXpert;

‡ , antiretroviral therapy,

§ , cotrimoxazole preventive therapy.

**FIGURE 2:**
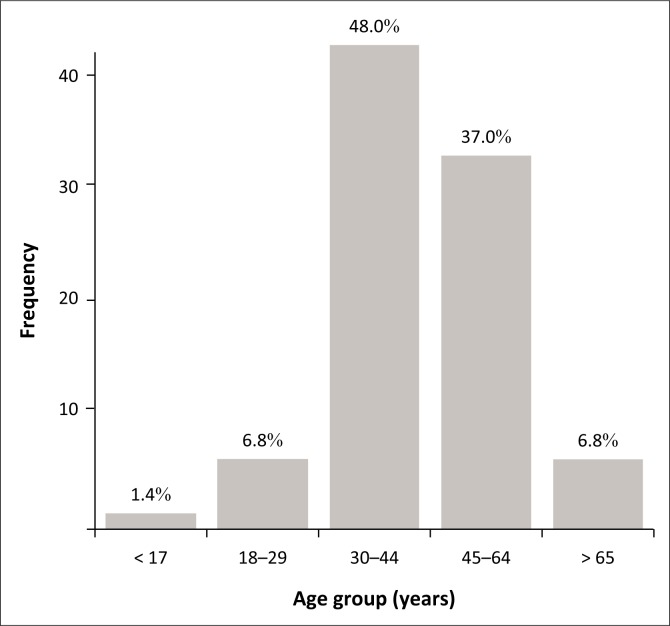
Death per age group among tuberculosis and HIV co-infected patients died before completing anti-TB treatment, Mahalapye Health District, January 2013 to December 2015.

In all, 73 (13.6%) TB and HIV co-infected patients died before completing anti-TB treatment. Figure 2 illustrates death per age group among TB and HIV co-infected patients who died before completing anti-TB treatment. Close to half (35 [47.95%]) of the cases were in the 30- to 44-year-old age group; and in the age group 45 to 64 years, 27 (37.0%) cases were observed. Male patients had the highest proportion of deaths compared with female patients (61.6% vs. 38.4%, *p* = 0.046).

In TB and HIV co-infected patients who died before completing anti-TB treatment, three-quarters (54 [74.4%]) were on ART. Only two patients were on ART at least 3 months prior to commencing anti-TB. Also, the majority (64 [87.7%]) of TB and HIV co-infected patients who died before completing anti-TB treatment were commenced on CPT.

**FIGURE 3:**
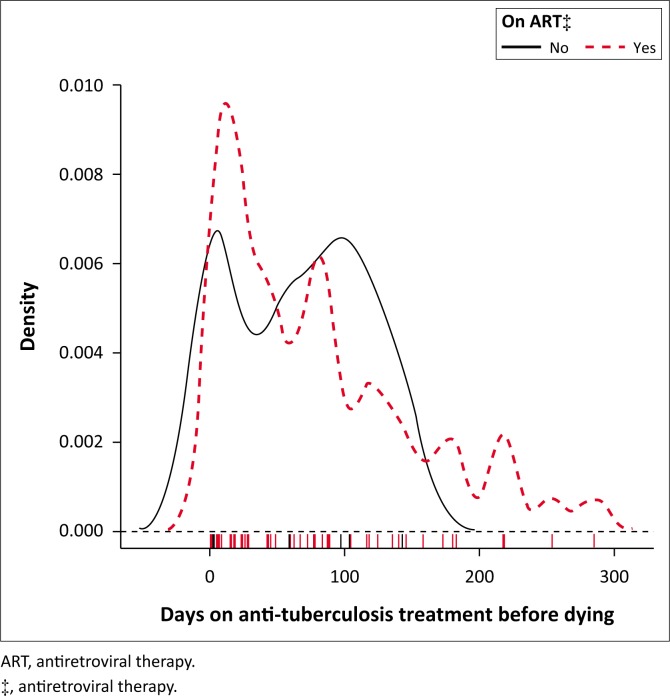
Kernel density plot, days on anti-tuberculosis treatment before death among tuberculosis and HIV co-infection cases, Mahalapye Health District, January 2013 to December 2015. On antiretroviral therapy or not.

**FIGURE 4:**
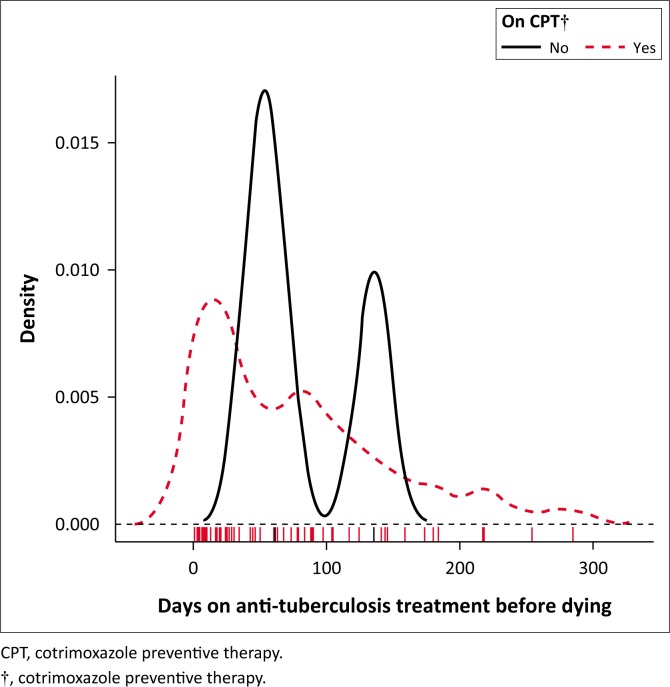
Kernel density plot, days on anti-tuberculosis treatment before death among tuberculosis and HIV co-infection cases, Mahalapye Health District, January 2013 to December 2015. On cotrimoxazole preventive therapy or not.

Figure 3 shows a bimodal density curve of death occurrence around the first and the third month in those who did not commence ART. Furthermore, a bimodal density curve of death occurrence was observed around the second and the fourth to fifth month in those who did not commence CPT (Figure 4).

## Discussion

This study established that all-cause mortality was 11.4% among TB patients regardless of their HIV status and 13.6% among TB and HIV co-infected patients in the MHD from 2013 to 2015. Similar findings (12.4%) were reported in a study in Limpopo Province in South Africa in 2003.^16^ However, a lower prevalence (5.3 %) of TB mortality was reported in Khayelitsha, South Africa in 2007. Nevertheless, in that study, 10.5% of TB patients were lost to follow-up and some of those patients could have died and the reported death rate could have been higher.

This study revealed that half of the cases (54.7%) had a TB and HIV co-infection. More effort should be made to test and record TB patients, as in 9.2% of the cases the HIV status was unknown. About 70.2% of pulmonary TB cases were bacteriologically confirmed by microbiology or GeneXpert. This proportion is higher compared to findings from other studies. For instance, a study conducted in Ethiopia in 2012 revealed a proportion of 33.0% of pulmonary TB that was bacteriologically confirmed after a culture in TB and HIV co-infected patients.^17^ The finding from this study of a high proportion of bacteriologically confirmed cases could be because of use of the GeneXpert in some cases, as a single GeneXpert test directly from sputum can detect 99% of smear-positive patients and more than 80% of patients with smear-negative disease.^18^

The majority of the TB and HIV co-infected patients were on ART (81.63%) or were initiated on CPT (87.2%) while taking anti-TB treatment in the MHD. This finding, although commendable, was still far from the 100% Botswana national standard.

This study found that the TB and HIV co-infected patients aged 30–44 years and 45–64 years had high mortality rates of 47.95% and 37.7%, respectively. Male patients had a high proportion of deaths compared to female patients (*p* = 0.046) in the MHD. Studies in the Southern African region by Mabunda et al. and Pepper et al. found that the most impacted age group was the economically active population between 25 and 54 years.^10,19,20^ The high TB mortality rate in middle adulthood could be because of the high prevalence of HIV infection in this age group compared to younger or older patients. With regards to sex, our study finding was dissimilar to that of Pepper et al., indicating an association between TB mortality and female gender in HIV-infected TB patients (adjusted odd ratio 1.24 [1.06–1.45], *p* = 0.01),^19^ while Alobu et al. did not find any difference in TB mortality between the two genders.^20^

This study indicated that the majority of TB and/or HIV patients died around the first month of their anti-TB treatment; the mortality rate reduced gradually from the first month for patients on ART or CPT. Although the determination of timing of ART and CPT may not have been feasible in this study, one could perceive the influence of ART and CPT on TB mortality. Three-quarters (74.4%) of TB and HIV co-infected patients who died before completing anti-TB treatment were on ART; only two patients were on ART at least 3 months prior to commencing anti-TB treatment. The majority (87.7%) of TB and HIV co-infected patients were on CPT prior to death in the MHD. Those who did not take ART had a high mortality rate during the first and the third month of their anti-TB treatment. Those who did not commence CPT had a high mortality rate during the second and the fifth month of their anti-TB treatment. Alobu et al. reported that the quasi-majority (91.5%) of TB mortality in their study occurred in the intensive phase.^21^ Although studies have reported a high TB mortality in the intensive phase of anti-TB treatment, there are limited investigations to establish the predictors of TB death in this phase.^17^ Immune reconstitution inflammatory syndrome could also contribute to TB mortality in the first 3 months of anti-TB treatment. Antiretroviral therapy needs at least 3 months to be protective in TB and HIV co-infected patients. Therefore, the implementation of a ‘treat all’ strategy could help to start patients on ART in time and reduce mortality in such patients.^21^

We could not determine the influence of CD4 levels on mortality in TB and HIV co-infected patients as ETR and TB facility registers could not provide such information. Also, M-DHMT-ETR could not provide accurate information on the number of patients diagnosed with pulmonary TB using GeneXpert, a valuable diagnostic tool with a high sensitivity in TB and HIV co-infected patients.

We recommend a community-oriented primary healthcare (COPC) based approach to detect both HIV and TB early. The ‘treat all’ approach should also be COPC based rather than health institution based as it is today in many countries, including Botswana. Community-oriented primary healthcare to reinforce TB contact tracing and early TB detection using lipoarabinomannan in urine in combination with the traditional sputum smear^22^ and GeneXpert testing may contribute to reduction of TB mortality. Prompt prescription of CPT and ART to reach a 100% uptake may also contribute to a decrease in deaths.

As the ‘treat all’ approach was introduced in Botswana in 2016, a study that looks into the period after its implementation is needed to assess whether it has an impact in the TB and/or HIV mortality in our setting.

Also, it was observed that TB mortality (11.4%) among TB patients regardless of their HIV status was still high compared to the 2025 WHO target; there is a need to identify factors that influence such mortality and address them.

## Conclusion

This study established that all-cause mortality among TB patients in the MHD was 11.4%, and TB and HIV co-infected patients had a TB mortality of 13.6%. The mortality rate of TB patients was still high compared to the WHO target of 6.5% by 2025. The majority of the TB and HIV co-infected patients were on ART (81.63%) or CPT had been initiated (87.2%). A high mortality rate was observed during the first and the third month in those who did not take ART and the second and the fifth month in those who did not commence CPT. Further study is needed to clarify this matter.
